# 

**Published:** 1899-11

**Authors:** 


### PERSONALS.

Dr. C. A. Dohan, of Darling, Pa., was a winner of a prize in the saddle-class at the Bryn Mawr horse show.

Dr. H. W. Stedman, of Barton, Vt., has removed to Springfield, Mass., where he will continue his professional work.

Dr. H. G. Black, of Wilkesbarre, Pa., was a visitor to the "City of Brotherly Love" the latter part of October.

Dr. H. L. Casey has returned to Frankfort, Kentucky, where he will resume practice after a sojourn in the fields of army service.

Dr. Castor, recently of the Bureau staff at Buffalo, N. Y., will do the work of ante-mortem or yard-inspection at Indianapolis, Ind.

Dr. and Mrs. H. S. Drake, of Leesburg, Va., were visitors to the National Export Exposition at Philadelphia in September.

Dr. A. E. Gruber, of Richlandtown, Pa., was a visitor to the JOURNAL office in September.

Dr. J. O. F. Price, Bureau of Animal Industry, has reported for duty at East St. Louis, Illinois.

Dr. Andrew Smith, of Toronto, Canada, will be one of the judges of hackneys at the National Horse Show in New York.

Dr. James B. Rayner, of West Chester, Pa., was a visitor to the JOURNAL office in September.

Dr. W. H. Dalrymple, of Baton Rouge, in an excellent article on "Beef Raising in Louisiana," gives some excellent advice to those who would enter this field with the hope of success.

Dr. Hiram P. Eves, of Wilmington, Del., is a lover of the trotter, and likes to try the mettle of his own fast goers at the race-tracks, holding the ribbons in person.

Dr. A. F. Schrieber, of Philadelphia, has so far completed his vocal training as to assure the members of the State Association at their annual gathering in March a rich entertainment of song and praise.

Dr. W. H. Fry, of Pine Grove Mills, was a visitor to New York in September to join in the home-coming reception of Admiral Dewey.

Dr. A. G. Kern, of Knoxville, Tenn., has taken up his medical studies at the Department of Medicine in the University of Pennsylvania.

Dr. R. G. Webster, of Salem, N. J., was a visitor to Philadelphia and the JOURNAL office in October.

Dr. Leonard Pearson, State Veterinarian of Pennsylvania, read a paper before the convention of livestock sanitary boards convened in Chicago in October.

Dr. Leonard Pearson was a visitor at Pittsburg in October, there to join with local veterinarians in the examination of a number of cattle condemned for tuberculosis, part of a herd of one of the State institutions in the western district.

Dr. James T. McAnulty, of Philadelphia, fills the rôle of Scientific Director to the Master Horseshoers' Protective Association.

Dr. Peters, of Massachusetts ; Dr. Pearson, of Pennsylvania ; Dr. Eisenman, of Kentucky; and Dr. Reynolds, of Minnesota, were in attendance at the recent meeting of the Interstate Association of Livestock Sanitary Boards at Chicago.

Dr. J. L. Clark, formerly of Stratford, Ontario, has recently located at Russell, Manitoba, where he finds the climate much more congenial to his impaired health.

Dr. A. C. Potteiger, of Selinsgrove, Pa., and two members of his family were bitten by a rabid dog of his own in October, and at once repaired to New York for treatment with the antirabific serum.

Dr. Jose T. Hernsheim, of Fargo, North Dakota, has recently located at Kenosha, Wisconsin.

Dr. G. E. Noble, of Osage, Iowa, is much interested in the breeding of high-class horses, St. Bernard and collie dogs, and numbers in his breeding stud many excellent sires.

Dr. H. Clay Glover, of New York City, was the judge of the setter classes at the Philadelphia dog show in November.

Dr. J. W. Elder, of Seaforth, Ontario, has succeeded to the practice of Dr. J. L. Clark, formerly of Stratford, Ontario.

Dr. Harry Walter, of Kirksville, Missouri, has again returned East, and will resume investigations in physiology and psychology under more advantageous circumstances.

Dr. E. A. A. Grange, of Detroit, Michigan, is now a resident of New York City and engaged in special work in connection with the Henry Bergh Society for Prevention of Cruelty to Animals.

Dr. Joseph Orr, who for a number of years was located at Baden, is now at Stratford, Ontario, doing a lucrative practice.

Dr. Oscar Seeley, of Philadelphia, is a student of horse form and action, and is pursuing a special line of investigation in the various breeds and types of horses used among Philadelphia's varied commercial interests.

Dr. Harvey F. Kaufman, of Hegins, Pa., graduate of the Ontario Veterinary College, class of '99, is completing his course at the United States Veterinary College.

Dr. S. S. Buckley, of College Park, Md., recently found one to his liking in the "Prairie State," and will no longer travel alone in the world.

Dr. A. S. DeWolf, of the Bureau of Animal Industry, has been transferred from Kansas City to Chicago.

Dr. D. McEachran, of Montreal, Canada, has just returned from a trip abroad.

Dr. R. S. Huidekoper made hurried visits to the Kansas City, Chicago, and McKillip Veterinary Colleges in connection with his Western trip.

Dr. A. A. Holcombe, of Cheyenne, Wyoming, has become an attaché of the Bureau of Animal Industry and stationed at Chicago.

Dr. George A. Knapp, formerly of Netherwood, N. Y., has now located at Millbrook, N. Y.

Dr. P. O. Koto, of Forest City, Iowa, a graduate of the Chicago Veterinary College, class of '95, is a candidate on the Republican legislative ticket of Iowa.

Dr. A. H. Balliet, of Ballietsville, Pa., has recently become the owner of several very promising youngsters in the trotting class.

The Buffalo *Horse World* said, in a recent issue, of "Creeping Flower," 2.22½, owned by Editor Gill, of this JOURNAL, that the above mark is no indication of her speed, and that she can step a half mile straightaway in 1.03. Surely a fast pacemaker for an editor to drive.

Dr. L. L. Cheney, V.M.D., graduate of the Veterinary Department of the University of Pennsylvania, class of '99, has permanently located at Augusta, Georgia, having succeeded to the practice of Dr. A. C. Walls.

Dr. D. E. Salmon, of the Bureau of Animal Industry was in attendance at the meeting of the State Livestock Sanitary Boards at Chicago in October.

Dr. Charles S. Gelbert, of Scranton, Pa., was a visitor to Philadelphia early in November. He cannot refrain from taking a shy at the football now and again, at which post he has won many honors.

